# Enrichment of Rare Variants in Nuclear-Encoded Mitochondrial Metabolism Genes in Patients with Early-Onset or Familial Parkinson’s Disease

**DOI:** 10.3390/genes17040472

**Published:** 2026-04-17

**Authors:** Gaber Bergant, Vesna M. van Midden, Polina Tsygankova, Dorian Laslo, Valentino Rački, Dejan Georgiev, Eliša Papić, Marija Branković, Milena Janković, Marina Svetel, Nataša Teran, Natasa Dragasević Misković, Igor Petrović, Aleš Maver, Ivana Novaković, Zvezdan Pirtošek, Martin Rakuša, Vladimira Vuletić, Borut Peterlin

**Affiliations:** 1 Clinical Institute for Genomic Medicine, University Medical Center Ljubljana, 1000 Ljubljana, Slovenia; 2 Department of Neurology, University Medical Center Ljubljana, 1000 Ljubljana, Slovenia; 3 Department of Neurology, Faculty of Medicine, University of Rijeka, 51000 Rijeka, Croatia; 4 Artificial Intelligence Lab, Faculty of Computer and Information Sciences, University of Ljubljana, 1000 Ljubljana, Slovenia; 5 Department for Clinical Genetics, University Children’s Hospital, 11000 Belgrade, Serbia; 6 Neurology Clinic, University Clinical Center of Serbia, 11000 Belgrade, Serbia; milena.jankovic.82@gmail.com; 7 Neurology Clinic, Faculty of Medicine, University of Belgrade, 11000 Belgrade, Serbia; 8 Institute of Human Genetics, Neurology Clinic, Faculty of Medicine, University of Belgrade, 11000 Belgrade, Serbia; 9 Department of Neurology, Faculty of Medicine, University of Ljubljana, 1000 Ljubljana, Slovenia; 10 Division of Neurology, University Medical Centre Maribor, 2000 Maribor, Slovenia

**Keywords:** Parkinson’s disease, mitochondrial metabolism, mitochondrial variants, mutation burden analysis

## Abstract

**Introduction**: Parkinson’s disease (PD) is a prevalent neurodegenerative disorder, with several proposed pathogenic mechanisms. Given the established role of mitochondrial dysfunction in PD, this study seeks to investigate the enrichment of rare genetic variants tied to mitochondrial metabolism in cases of early-onset and familial PD. **Methods**: We performed a retrospective analysis on 248 early-onset and familial PD patients and 1622 control individuals. We assessed both pathway-level and gene-level burden of germline rare variants detected using exome sequencing in 467 nuclear genes related to mitochondrial metabolism. **Results**: Gene-set mutation burden analysis indicated an increased burden in genes associated with mtDNA maintenance. In addition, gene-level analysis identified a possible association between PD and rare variant burden in 14 mitochondrial metabolism-related genes under dominant or recessive inheritance models. **Conclusions**: Our findings support a potential contribution of rare germline variants affecting mitochondrial metabolism to the susceptibility in early-onset and familial PD.

## 1. Introduction

Parkinson’s disease (PD) is the second most common neurodegenerative disease and as such represents a growing public health challenge. It affects 9.5 out of every 1000 people over 65 years of age [1]. The hallmarks of the disease are characteristic motor symptoms caused by loss of dopaminergic neurons in substantia nigra pars compacta [1]. Pathologic findings include aggregations of alpha-synuclein in Lewy bodies in affected brain regions [1]. While misfolding of alpha-synuclein is considered central to the pathogenesis of PD, several biological processes have been shown to contribute to neuronal cell death in PD including mitochondrial dysfunction [2].

Mitochondrial dysfunction was established as a cause of dopaminergic neuronal loss after complex I inhibitors were found to directly cause parkinsonism [2]. The involvement of mitochondrial dysfunction in PD was further supported by the finding of respiratory complex I dysfunction in tissues from both sporadic and genetic PD patients [3]. Furthermore, multiple genes associated with monogenic autosomal dominant and recessive PD have also been implicated in mitochondrial dysfunction including *SNCA*, *LRRK2*, *CHCHD2*, *GCH1*, *VPS35*, *PINK1* and *PRKN* genes [2].

Mitochondrial dysfunction in PD can be conceptualized through three interconnected mechanisms. First, α-synuclein exerts direct mitochondrial toxicity [4]. Pathogenic SNCA associates with mitochondrial membranes, disrupting membrane integrity, impairing protein import, and inhibiting respiratory chain activity—particularly complex I. These effects amplify reactive oxygen species (ROS) production, leading to bioenergetic failure and oxidative damage, and establishing a self-reinforcing cycle linking protein aggregation with mitochondrial stress [5].

Second, nuclear-encoded PD-associated genes converge on mitochondrial homeostasis through both direct and indirect pathways. Genes such as PINK1 and PRKN regulate mitophagy, enabling the selective removal of dysfunctional mitochondria [6]. On the other hand, CHCHD2 supports respiratory chain integrity [4]. In parallel, a recent meta-analysis identified genes that primarily affect vesicular trafficking and lysosomal function [7]. LRRK2, TMEM175, and VPS35 play an essential role in mitochondrial turnover. Disruption across these systems leads to accumulation of damaged mitochondria, increased oxidative stress, and heightened neuronal vulnerability.

The mitochondrial genome plays a significant role in selective susceptibility. Human mitochondrial DNA encodes 13 subunits of the respiratory chain, seven of which are part of complex I [8]. This provides a mechanistic explanation for the frequent dysfunction of complex I observed in PD. Changes in mitochondrial DNA can impair oxidative phosphorylation, increase ROS production, and activate pathways leading to cell death [4].

Despite significant knowledge of nuclear-encoded mechanisms, the role of mitochondrial DNA remains poorly understood. Since mitochondrial DNA is crucial for encoding essential components of complex I and for regulating cellular energy production, a thorough investigation of its variation and function is a vital step toward understanding the mechanisms of PD.

The common disease—rare variant hypothesis—argues that susceptibility to common diseases can be increased by multiple rare variants [9]. While research on monogenic PD and GWAS studies are producing clinically relevant results [10,11], they are not designed to detect multiple rare variants that have the potential to collectively alter the function of a gene’s protein product, or a biological pathway involved in the pathogenesis of PD. Functional studies of fibroblasts in sporadic PD patients which showed a subset of patients with impaired mitochondrial function could indicate the existence of germline genetic variants in mitochondrial metabolism-associated genes causing a dysfunction [12]. Recently Billignsley et al. showed that multiple low-effect variants in genes associated with mitochondrial function can lead to a significantly increased risk for PD [13]. They were able to identify 14 genes associated with mitochondrial function that were linked to PD risk [13]. Furthermore, another study found an enrichment of rare variants in a subset of genes involved with mitochondrial replication and repair, leading to the hypothesis that this enrichment contributes adversely to mitochondrial homeostasis [14].

The aim of our study is to analyze the germline rare variant burden in nuclear genes associated with mitochondrial metabolism in patients with early-onset (EOPD) or familial PD. We selected these groups because of their potentially substantial genetic contribution to disease risk. Our hypothesis is that the burden is higher in these PD patients compared to the control individuals. The higher variant burden could indicate dysfunction leading to the impaired mitochondrial function contributing to the development of PD.

## 2. Methods

We performed a retrospective analysis of 248 Slovenian, Croatian and Serbian patients with Parkinson’s disease consecutively referred to Clinical Institute of Medical Genetics, University Medical Center Ljubljana, Slovenia, for whole exome sequencing in the period between July 2014 and January 2021. All patients were counseled and consented to their de-identified samples and data being used for research purposes. The control group comprised 1622 Slovenian, Croatian and Serbian samples from unrelated patients referred to our institution for diagnoses unrelated to Parkinson’s disease, neurodegenerative or mitochondrial disorders.

Sequencing was performed using a standardized set of protocols. Exome sequences were captured using Twist Human Core Exome (Twist Bioscience, San Francisco, CA, USA), TruSight One, Nextera Coding Exome and IDT Exome capture kits (all manufactured by Illumina, San Diego, CA, USA) or Agilent SureSelect Human All Exon v7 capture kit (manufactured by Agilent Technologies, Santa Clara, CA, USA), which was followed by sequencing on Illumina NextSeq 550 and Illumina NovaSeq 6000 platforms. Sequencing data analysis and variant calling was performed using a custom genomic data analysis pipeline based on the Genome Analysis Toolkit (GATK) Best Practices workflows [15]. Briefly, alignment to the hg19 human genome reference assembly was performed using Burrows–Wheeler (BWA) aligner, with the duplicate sequences removed using Picard Tools MarkDuplicates (version 4.0.1.1) [16,17]. Base quality score recalibration, variant calling, variant quality score recalibration and variant filtering were performed using elements of the GATK toolset [15]. Join genotyping, variant storage, annotation and filtration was based on GATK (version 4.2) and VEP (version 102) software [15,18]. The cutoff frequency for rare variants was 5% in the gnomAD database v2.1 [19].

Sequencing quality control (QC) was performed on all samples and included assessment of sequencing depth (median sequencing depth over 60×), molecular sex match, heterozygosity ratio and rate of coverage (over 98% of regions sequenced with at least 20× coverage). We also performed quality control on the individual variants with required variant quality over 20 and variant coverage over 10 for inclusion in further analysis.

We included 248 PD patients who had (1) a confirmed clinical PD diagnosis based on United Kingdom Parkinson’s Disease Society Brain Bank clinical diagnostic criteria (UKPDSBB) [20], (2) either early onset sporadic PD (onset before 50 years of age) or familial PD with affected 1st or 2nd degree relatives, and (3) no clinically relevant variants associated with Parkinson’s disease or other neurodegenerative diseases found in exome sequencing data. Patients with secondary parkinsonism, atypical parkinsonian syndromes, or alternative neurological diagnoses were not considered for inclusion in the study. One sample failed the sequencing QC analysis, leaving us with 247 PD patients. Comprising the control group were 1622 patients referred to our institution with a diagnosis unrelated to PD, any neurodegenerative or mitochondrial disorders.

Genes associated with mitochondrial metabolism were defined using curated gene sets from the Genomics England PanelApp clinical diagnostic panels for mitochondrial disease. Specifically, we selected the following panels: mitochondrial disorders (v2.4), Possible mitochondrial disorder—nuclear genes (v1.17), mitochondrial disorder with complex I deficiency (v1.2), complex II deficiency (v1.2), complex III deficiency (v1.2), complex IV deficiency (v1.2), complex V deficiency (v1.2), mitochondrial DNA maintenance disorder (v1.2), and mitochondrial liver disease (v1.2). We included only nuclear genes with green expert review ratings. This resulted in a final set of 467 genes.

Gene-level enrichment analysis of rare variants was then performed using TRAPD software with Benjamini–Hochberg procedure to control the false discovery rate. Genes with less than three qualifying variants in the patient or the control cohorts were not considered in the analysis, as the association could be contributed to a single variant. Analysis was performed separately for rare missense and loss-of-function (frameshift, stopgain and canonical splice donor and acceptor variants) variants. Additionally, we performed a gene-set burden analysis of rare missense variants in each included Genomics England PanelApp gene panel representing mitochondrial metabolism subpathways. Finally, we also included the complete gene set in the gene-set burden analysis with variants separated by variant type.

## 3. Results

Following joint genotyping and quality control we obtained 6036 rare missense and 648 rare loss-of-function variants in our cohort of 247 PD individuals and 1622 controls. The PD cohort had a mean age of 62.5 years and included 149 males and 98 females. According to the study inclusion criteria, 97 patients had early-onset PD, 105 had familial PD, and 45 fulfilled both criteria. The control cohort had a mean age of 36.5 years and included 843 males and 779 females.

Gene-set missense mutation burden analysis yielded one gene-set passing the statistical significance threshold of *p* < 0.05 which included genes associated with mitochondrial DNA (mtDNA) maintenance. Mutation burden analysis in other gene-sets, including the gene-set of all genes associated with mitochondrial metabolism included in this study, did not yield statistically significant results.

Gene-level burden analysis yielded the results for 102 and three genes in missense and loss-of-function categories, respectively. For detailed results see the Supplementary Materials. Other genes did not pass the criteria of three or more variants per gene in each cohort and were eliminated from subsequent analysis.

Missense variant burden testing yielded eight and six genes passing the statistical significance threshold of *p* < 0.05 under the dominant model and recessive models, respectively. Following false discovery rate correction, no genes passed the significance threshold under any model.

Loss-of-function variant burden test did not yield any genes passing the statistical significance threshold. Detailed results are presented in Table 1.

## 4. Discussion

In this retrospective analysis of patients with early-onset or familial PD, we assessed the burden of rare variants in genes associated with mitochondrial metabolism. We observed nominal (uncorrected) increases in rare-variant burden in eight genes under a dominant inheritance model (PNPT1, FBXL4, AARS2, MRPS2, NDUFB8, SLC25A19, PUS1, and ATPAF2) and in six genes under a recessive inheritance model (DLAT, MRPL3, TOP3A, SDHA, HLCS, and FDXR).

The gene candidates identified in this study may implicate mitochondrial dysfunction at several levels of mitochondrial biology, ranging from respiratory chain function to mitochondrial gene expression and genome maintenance. NDUFB8 and SDHA encode subunits of complexes I and II of the oxidative phosphorylation (OXPHOS) system, respectively, while ATPAF2 encodes an assembly factor required for complex V biogenesis [21]. In addition, disruption of mitochondrial translation-related pathways, including mitoribosomal proteins such as MRPL3 and MRPS2 [22], and genes involved in mitochondrial tRNA biology and RNA processing, such as AARS2, PUS1 [23,24] and PNPT1 [21], could impair synthesis of the 13 mtDNA-encoded respiratory chain subunits. Such defects would be expected to affect complexes I, III and IV most directly and could result in combined OXPHOS deficiency.

In addition, DLAT is a core component of the pyruvate dehydrogenase complex, while SLC25A19 encodes the mitochondrial thiamine pyrophosphate transporter and may therefore influence pyruvate dehydrogenase-dependent metabolism [25,26]. Variants in TOP3A and FBXL4 may likewise be relevant to mitochondrial genome homeostasis and could, in principle, contribute to impaired mtDNA maintenance [27,28]. Taken together, these observations suggest that several of the candidate genes converge on pathways relevant to mitochondrial energy metabolism, with a possible effect on complex I-associated vulnerability.

Of these, PNPT1, AARS2, and NDUFB8 have been implicated in the prior literature in PD-relevant phenotypes, including parkinsonism within multisystem mitochondrial disorders [3,24,29,30].

Using gene-set burden analysis, we further found that the mtDNA maintenance gene set showed a higher rare-variant burden in our early-onset and familial PD cohort compared with controls. Mitochondrial DNA (mtDNA) maintenance is governed by a network of proteins involved in mtDNA replication, mitochondrial dynamics (including fusion and fission), and regulation of the mitochondrial deoxynucleotide pool. Unlike nuclear DNA, mtDNA is continuously replicated and is not regulated by the cell cycle. Disruption of mtDNA maintenance can lead to impaired mtDNA integrity, manifesting as multiple deletions or duplications, as well as a reduction in mtDNA copy number (mtDNA depletion). These defects result in compromised oxidative phosphorylation, cellular energy deficiency, and ultimately cell death [31].

Dölle and colleagues demonstrated that, in healthy individuals, mtDNA copy number in substantia nigra neurons increases with age, reflecting dynamic regulation of mitochondrial genome content [32]. In contrast, this adaptive increase is absent in patients with Parkinson’s disease (PD) [32]. Consistent with these findings, Pyle et al. reported a significant reduction in mtDNA copy number in post-mortem substantia nigra tissue from PD patients compared with controls, supporting the notion that impaired regulation of mtDNA content is a characteristic feature of the disease. As the neuronal burden of mtDNA mutations was comparable between PD patients and healthy controls, these observations suggest that dysregulation of mitochondrial DNA homeostasis—rather than an increased mutational load—underlies respiratory chain deficiency in affected neurons, thereby contributing to PD pathogenesis [33].

This study has several strengths, foremost among them the comprehensive analysis of nuclear genes involved in mitochondrial metabolic pathways. However, it is not without limitations. The primary constraint lies in the size of the patient cohort; increasing the cohort could capture a broader range of genomic variability, potentially identifying more genes that meet the criteria for analysis. Furthermore, we did not perform systematic variant-level functional or pathogenicity prediction analyses and our findings should be interpreted at the level of gene-set and gene burden rather than as evidence for the functional impact of individual variants. Additionally, although the genetic differences among the populations included in the study are anticipated to be minimal due to shared historical and geographical contexts, the possibility of introducing population-based bias cannot be entirely excluded.

Given prior evidence of dysregulated mtDNA homeostasis in PD and the signal observed in our mtDNA maintenance gene-set analysis, strategies aimed at enhancing mtDNA maintenance warrant further investigation as potential disease-modifying approaches. The stimulation of the mtDNA maintenance could improve mitochondrial function, alleviate energy deficits, and reduce neuronal loss, thus addressing a core aspect of PD pathology [34].

In conclusion, we observed a gene-set signal for mtDNA maintenance and nominal, hypothesis-generating gene-level burden differences across 14 mitochondrial metabolism genes. Together, these results are consistent with a model in which the cumulative effect of multiple rare variants may contribute to PD susceptibility and further highlight mtDNA maintenance as a mechanistically relevant pathway for follow-up in larger, well-matched cohorts.

## Figures and Tables

**Table 1:** Statistically significant results of missense variant burden analysis following both dominant and recessive inheritance models.

Gene	Case Count HET	Case Count CH	Case Count HOM	Case Total AC	Control Count HET	Control Count HOM	Control Total AC	*p*-Value DOM	*p*-Value REC	*p*-Value DOM Adjusted	*p*-Value REC Adjusted
PNPT1	8 (3.3%)	0 (0%)	0 (0%)	8 (3.3%)	12 (0.7%)	0 (0%)	12 (0.7%)	0.0025	NS	NS	NS
FBXL4	21 (8.5%)	0 (0%)	3 (1.2%)	27 (10.9%)	83 (5.1%)	0 (0%)	83 (5.1%)	0.0048	NS	NS	NS
AARS2	34 (13.8%)	3 (1.2%)	0 (0%)	38 (15.4%)	142 (8.8%)	0 (0%)	142 (8.8%)	0.0106	NS	NS	NS
MRPS2	16 (6.5%)	0 (0%)	0 (0%)	16 (6.5%)	51 (3.1%)	0 (0%)	51 (3.1%)	0.0111	NS	NS	NS
NDUFB8	4 (1.6%)	0 (0%)	0 (0%)	4 (1.6%)	6 (0.4%)	0 (0%)	6 (0.4%)	0.0327	NS	NS	NS
SLC25A19	4 (1.6%)	0 (0%)	0 (0%)	4 (1.6%)	6 (0.4%)	0 (0%)	6 (0.4%)	0.0327	NS	NS	NS
PUS1	13 (5.3%)	0 (0%)	1 (0.4%)	15 (6.1%)	50 (3.1%)	0 (0%)	50 (3.1%)	0.0352	NS	NS	NS
ATPAF2	27 (10.9%)	0 (0%)	1 (0.4%)	29 (11.7%)	128 (7.9%)	0 (0%)	128 (7.9%)	0.0485	NS	NS	NS
DLAT	8 (3.3%)	7 (2.8%)	0 (0%)	15 (6.1%)	115 (7.1%)	0 (0%)	115 (7.1%)	NS	0.0016	NS	NS
MRPL3	15 (6.1%)	5 (2.0%)	0 (0%)	20 (8.1%)	92 (5.7%)	0 (0%)	92 (5.7%)	NS	0.0056	NS	NS
TOP3A	15 (6.1%)	4 (1.6%)	0 (0%)	19 (7.7%)	87 (5.4%)	0 (0%)	87 (5.4%)	NS	0.0218	NS	NS
SDHA	44 (17.8%)	17 (6.9%)	1 (0.4%)	72 (29.1%)	326 (20.1%)	0 (0%)	326 (20.1%)	NS	0.0222	NS	NS
HLCS	18 (7.3%)	5 (2.0%)	0 (0%)	25 (10.1%)	123 (7.6%)	0 (0%)	123 (7.6%)	NS	0.0282	NS	NS
FDXR	11 (4.5%)	2 (0.8%)	0 (0%)	13 (5.3%)	48 (3.0%)	0 (0%)	48 (3.0%)	NS	0.0476	NS	NS

Case count HET—number of case group individuals with heterozygous variants; case count CH—number of case group individuals carrying two or more qualifying variants; case count HOM—number of case group individuals with homozygous qualifying variants; case total AC—total variant count in case group individuals; control count HET—number of control group individuals with heterozygous variants; control count HOM—number of control group individuals with homozygous qualifying variants; control total AC—total variant count in control group individuals; *p*-value DOM—*p*-value calculated using two-sided Fisher’s exact test under dominant inheritance model; *p*-value REC—*p*-value calculated using two-sided Fisher’s exact test under recessive inheritance model; *p*-value DOM-adjusted—adjusted *p*-value for dominant model using Benjamini–Hochberg correction; *p*-value REC-adjusted—adjusted *p*-value for recessive model using Benjamini–Hochberg correction; NS—not statistically significant (*p*-value > 0.05).

## Data Availability

The original contributions presented in this study are included in the article/Supplementary Materials. Further inquiries can be directed to the corresponding author.

## Supplementary Materials

The following supporting information can be downloaded at https://www.mdpi.com/article/10.3390/genes17040472/s1.
